# Wilkie's Syndrome as a Cause of Intestinal Obstruction and Chronic Abdominal Pain: A Case Report

**DOI:** 10.7759/cureus.87141

**Published:** 2025-07-01

**Authors:** Christian Ballardo Medina, Ana Karen Carrasco Gaxiola, Jaime Matus Rojas, Maria Valeria Figueroa Beltran, Rodolfo Lopez Hernandez, Jose Manuel Rocha Chavez

**Affiliations:** 1 General Surgery, Instituto de Seguridad y Servicios Sociales de los Trabajadores del Estado/Universidad Autónoma de Sinaloa (ISSSTE/UAS), Culiacán, MEX; 2 General Surgery, Instituto de Seguridad y Servicios Sociales de los Trabajadores del Estado (ISSSTE), Culiacán, MEX

**Keywords:** duodenojejunostomy, intestinal obstruction, strong procedure, superior mesenteric artery syndrome, wilkie's syndrome

## Abstract

Wilkie's syndrome (WS), or superior mesenteric artery (SMA) syndrome, is an extremely rare condition, with only a small number of cases reported. It is characterized by a picture of high intestinal obstruction, due to the compression of the third portion of the duodenum, as a consequence of a narrowing of the aortomesenteric angle (approximately <25°). We report the case of a woman in her fifth decade of life, who reports chronic abdominal pain and comes to the emergency department presenting with a picture of intestinal occlusion. During her management, an abdominal computed tomography (CT) scan is performed, with evidence of a decrease in the aortomesenteric angle and the aortomesenteric distance, with dilation of the first two duodenal and gastric portions. She underwent exploratory laparotomy, where a duodenojejunostomy was performed.

## Introduction

Wilkie's syndrome (WS) or superior mesenteric artery (SMA) syndrome or cylinder syndrome is characterized by gastrointestinal obstruction due to the compression of the anterior wall of the third portion of the duodenum due to the narrowing of the aortomesenteric arch. This was first described by Carl Von Rokitansky in 1861 and later by Wilkie in 1927 [1]. With a low frequency of presentation, just over 500 cases have been described, with a prevalence of 0.013%-0.3%, more frequent in women in a 2:1 ratio and in thin individuals between 10 and 39 years of age [1-3].

The loss of perivascular and retroperitoneal fat marks the origin of this pathology, which can be conditioned by congenital factors and pathologies that cause hypercatabolism or malnutrition, such as neoplasms, malabsorption syndromes, AIDS, trauma, burns, and multiple surgeries, with subsequent duodenal compression, which can be complete or partial and behave in a chronic or acute way, manifesting a diversity of symptoms [1,2].

The objective of this study is to conduct an extensive review of the international literature on the medical and surgical management of Wilkie's syndrome, as well as to present a clinical case of this pathology managed at our institution.

## Case presentation

**Figure 1:**
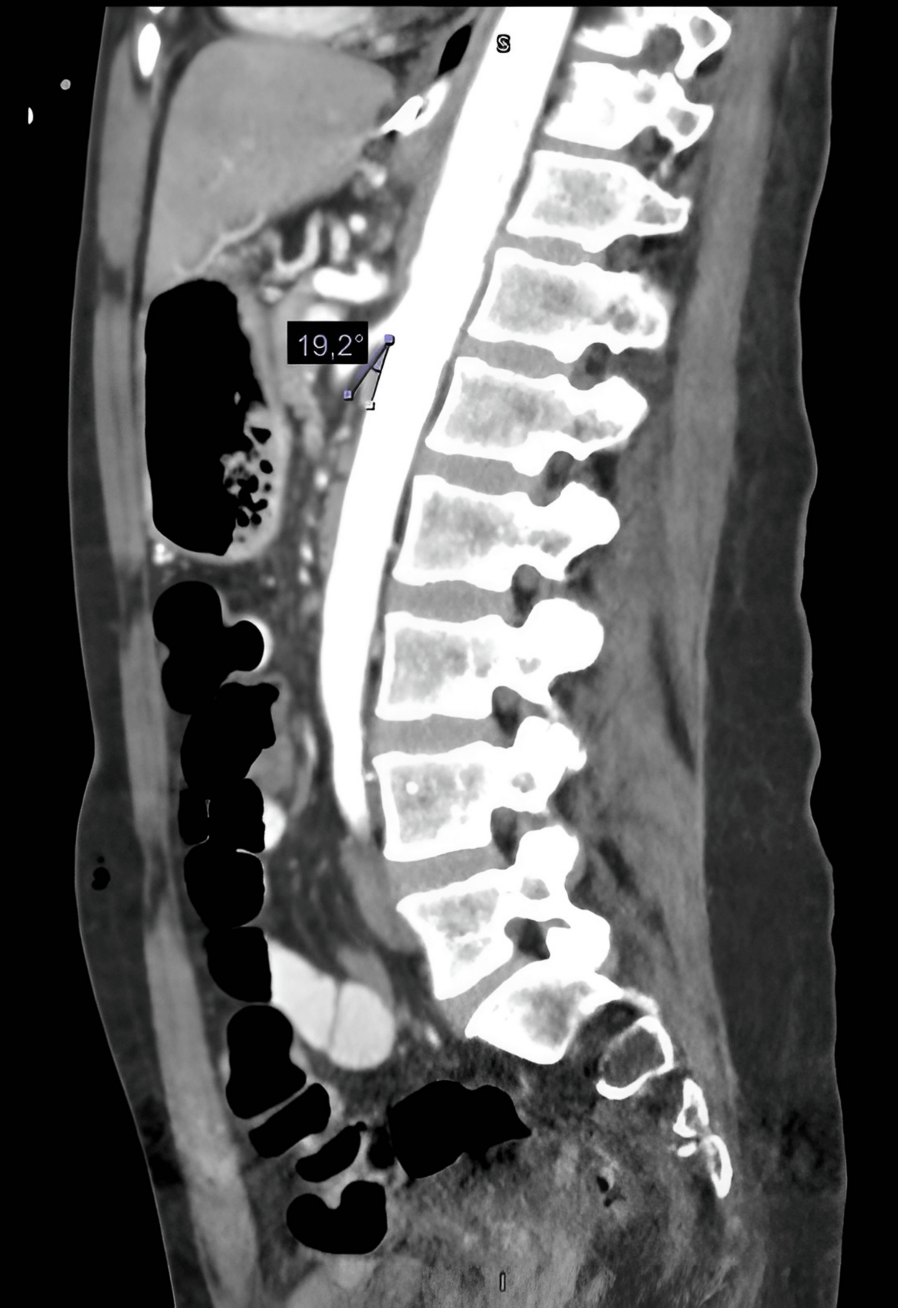
Sagittal CT scan. Measurement of the aortomesenteric arch (aortomesenteric angle measures 19.2°). CT: computed tomography

**Figure 2:**
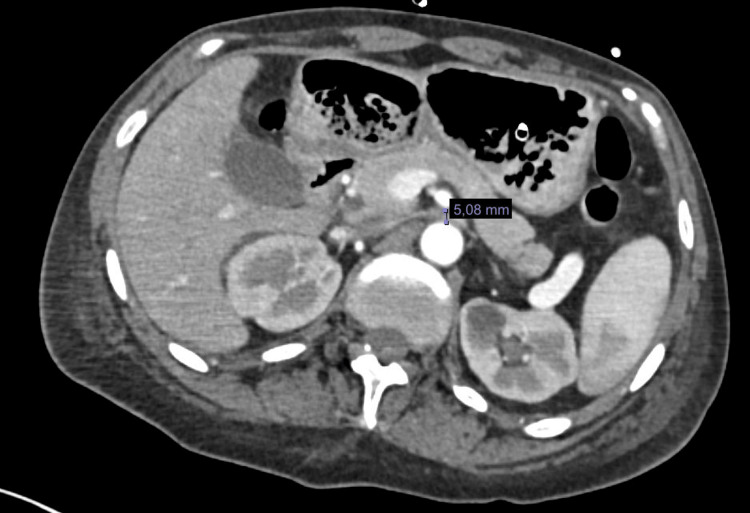
Axial CT scan. Measurement of aortomesenteric distance (distance of 5.08 mm). CT: computed tomography

**Figure 3:**
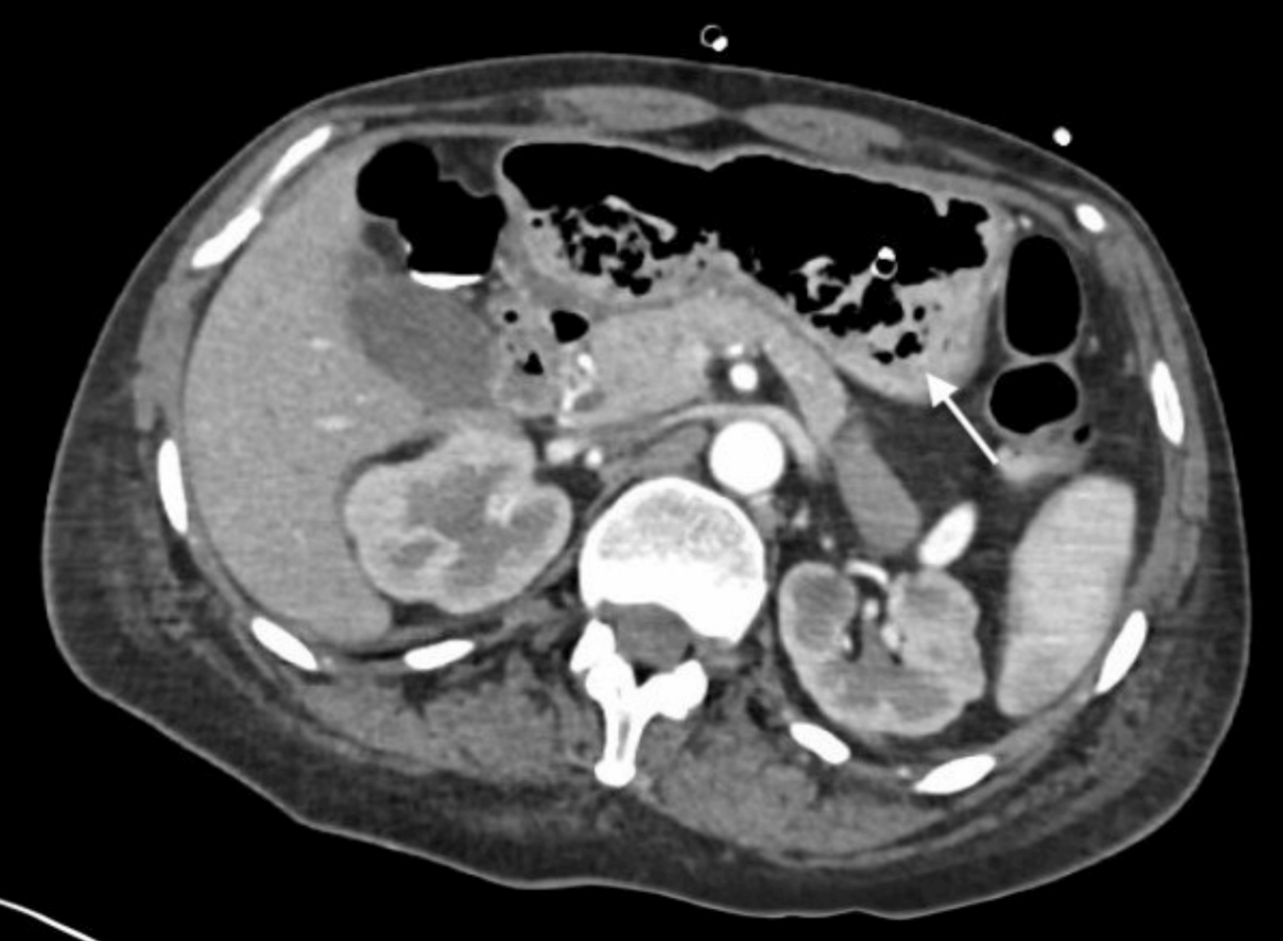
Gastric dilation and first and second duodenal portion dilation (marked by a white arrow).

**Figure 4:**
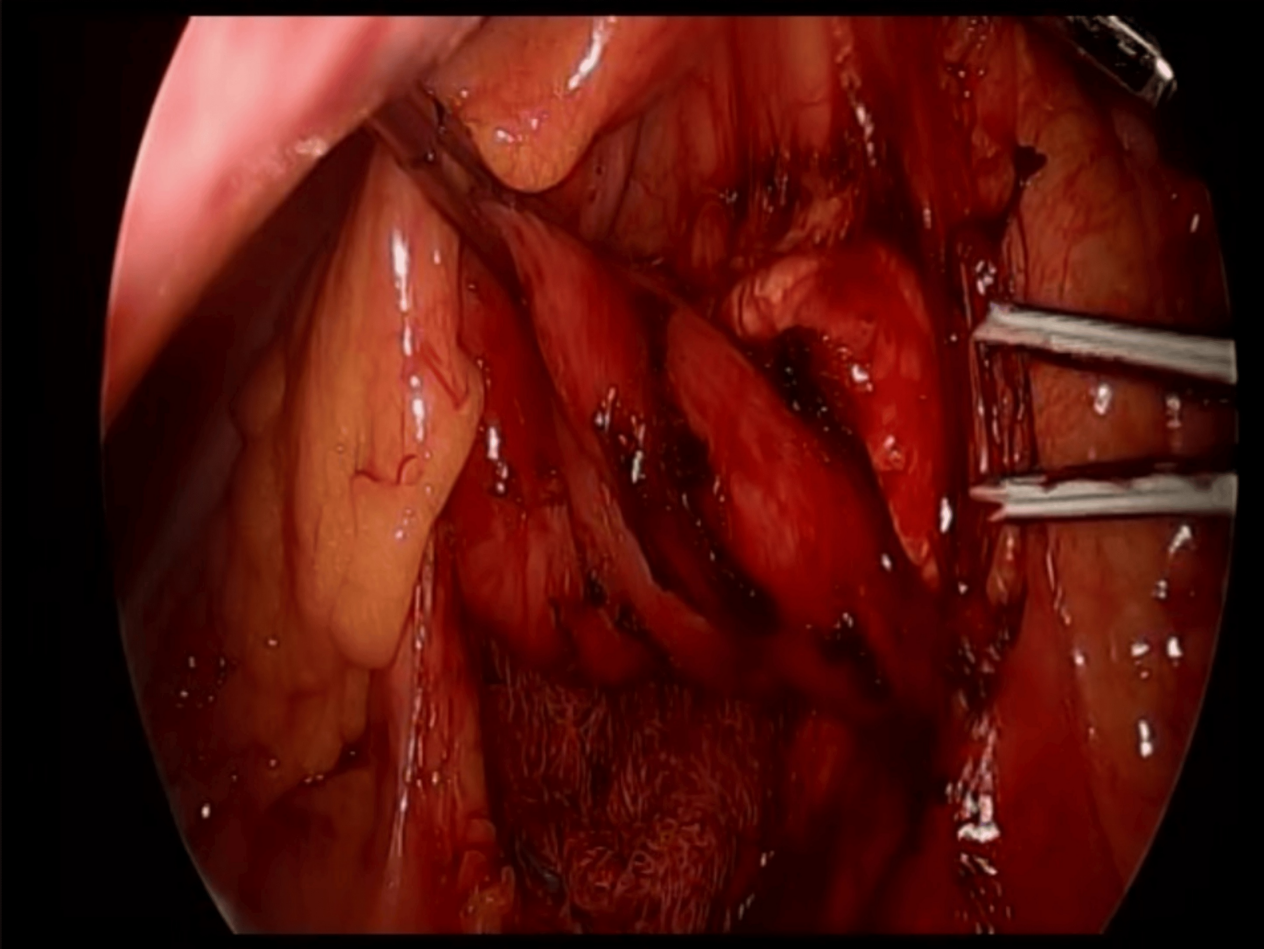
Duodenal release (Strong procedure).

**Figure 5:**
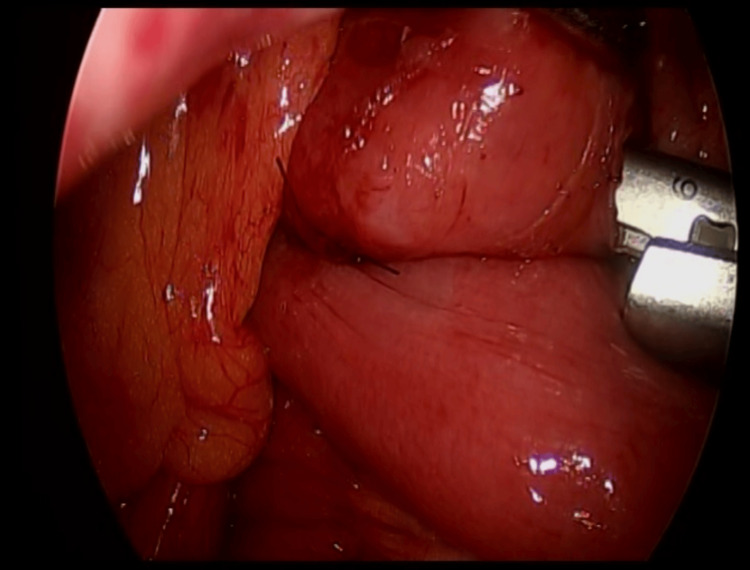
Side-to-side isoperistaltic duodenojejunostomy. Side-to-side isoperistaltic duodenojejunostomy with an Endo GIA stapler with 60 mm gold staples.

**Figure 6:**
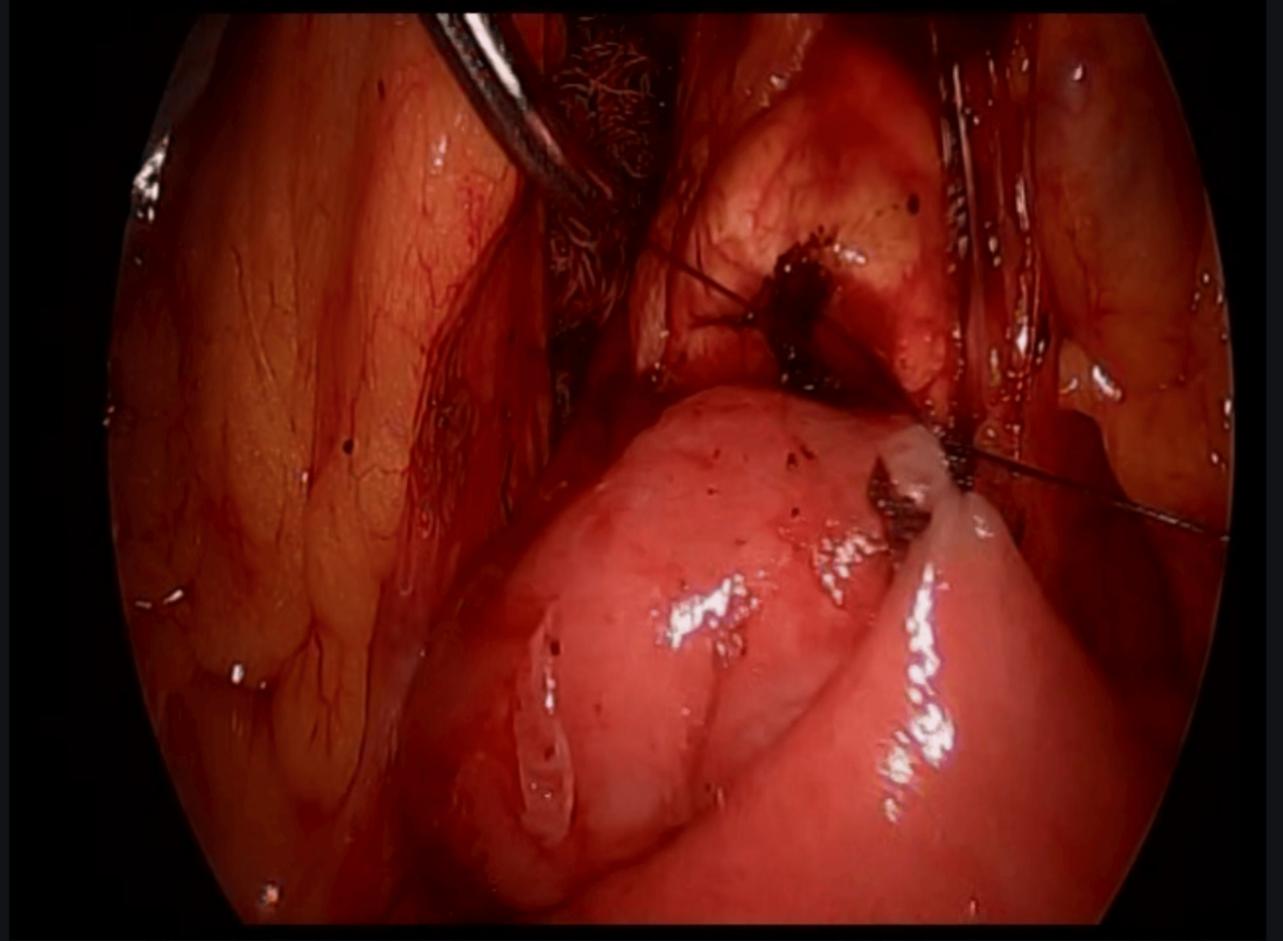
Side-to-side isoperistaltic duodenojejunostomy. Closure of the distal angle with monocryl 3-0 Cushing sutures.

We report the case of a 47-year-old woman with a positive chronic history of rheumatoid arthritis treated with methotrexate, tramadol/paracetamol, and etoricoxib. She reported allergies to hydrocortisone, prednisone, pregabalin, leflunomide, and nitrofurantoin and denied surgical history. She began her illness with nausea and vomiting of gastro-alimentary characteristics on three occasions after meals. This was accompanied by abdominal distension and abdominal pain, diffuse, poorly localized, and colicky type, which did not subside after taking butylhioscine. Physical examination revealed a distended, soft, and depressible abdomen, with pain on medium and deep palpation. Peristalsis was normoaudible, with no signs of peritoneal irritation. During her hospital stay, an abdominal computed tomography (CT) scan was performed, which showed an aortomesenteric angle of 19.2° (Figure 1) and an aortomesenteric distance of 5.08 mm (Figure 2), with dilation of the stomach and the first and second portions of the duodenum (Figure 3). Medical management was initiated, with nasogastric tube placement, parenteral nutrition (PN), and electrolyte management, and she was scheduled for diagnostic laparoscopy. During the procedure, the duodenal portions and the ligament of Treitz were identified and dissected with advanced bipolar energy, completing the Strong technique (Figure 4). Subsequently, it was decided to perform a side-to-side isoperistaltic duodenojejunostomy with an Endo GIA (Medtronic Minimally Invasive Therapies, Minneapolis, MN) stapler with 60 mm gold staples and the closure of the distal angle with monocryl 3-0 Cushing sutures (Figures 5, 6). In the postoperative course, a liquid diet was started at 72 hours with adequate tolerance; an abdominal CT scan with double contrast was performed to assess anastomosis permeability, without alterations. The nasogastric tube and Blake-type drain were removed, and she was discharged on the fifth postoperative day.

## Discussion

The diagnosis of this pathology becomes a true challenge for the surgeon. Its clinical presentation is nonspecific and sometimes intermittent, accompanied by epigastric pain (59%), abdominal distension, postprandial fullness, early satiety (32%), nausea (50%), and bilious, gastro-alimentary, or sometimes projectile vomiting, in its chronic presentation or acute exacerbation, which tends to improve when patients adopt a genupectoral position or left lateral decubitus, widening the aortomesenteric angle [1-4]. In our case, the patient presents with a clinical picture suggestive of high intestinal obstruction and classic symptoms of WS.

Despite the difficulty, a high clinical suspicion can be supported by various diagnostic studies. As an initial study, we have plain abdominal radiography, which will give us nonspecific data such as the dilation of the proximal duodenum and the absence of distal intestinal gas, typical of an occlusive picture. The esophagogastroduodenal series is a study of great utility, which can show duodenal dilation in the first or second duodenal portion, with or without gastric dilation, narrowing in the third duodenal portion, and delayed emptying of the barium contrast [1-5]. Upper endoscopy can confirm the obstruction at the level of the third duodenal portion and extrinsic compression [6]. CT and CT angiography or magnetic resonance imaging have become useful methods for evaluating duodenal compression, performing a measurement of the aortomesenteric arch, with an average measurement of 38°-65° and in WS reaching levels of <25°, causing a decrease between both vessels of <10 mm (10-28 mm average) [3,7]. In this patient, it was decided to perform CT with double contrast, looking for causes of mechanical intestinal obstruction; with a decrease in the aortomesenteric arch (19.1°) and the distance between the aorta and SMA, the distension of the first and second duodenal portion and gastric dilatation are evidenced.

Once the diagnosis is made, conservative management should be initiated, especially in acute presentations, with nasogastric tube placement for decompression, electrolyte management, and hypercaloric diet, preferably enteral and, if necessary, through a postpyloric tube, with the objective of increasing the patient's weight and the retroperitoneal fat pad; this management can be maintained for up to six weeks before considering a surgical procedure, with an approximate success rate of <71% [1,2,8,9]. Although surgical indications are not widely described, it is accepted that failure in medical management, long-term pathology with duodenal dilatation, and diseases associated with the stasis of biliary and gastric flow are indications for surgical management [8]. In our case, conservative management is initiated, due to the patient reporting the absence of improvement in the symptoms referred to; it is decided to prepare for diagnostic laparoscopy plus duodenojejunostomy.

Among the surgical alternatives, we had the following: the Strong procedure, gastrojejunostomy, and duodenojejunostomy (open or laparoscopic), with an approximate success rate of 96% [1,8,9].

The Strong procedure or a section of the ligament of Treitz, with the consequent mobilization of the duodenum, is an option widely used in pediatric patients, especially when they have previous surgeries, which presents an approximate failure rate of 25% [1,8].

Gastrojejunostomy is a procedure that has gradually ceased to be an adequate option for this type of patient, because, despite allowing gastric decompression, it does not resolve the duodenal occlusion, causing problems due to biliary reflux, adding to the fact that it is associated with blind loop syndrome [1,8,9].

In adults, the treatment of choice is duodenojejunostomy, introduced as a therapeutic option in patients with WS since 1910 and performed laparoscopically for the first time in the 1990s by Gersin and Heniford, with a success rate of 90%, which, when performed laparoscopically, presents with less postoperative pain, hospital stay, and complication rate [8-10]. The procedure performed on our patient was a section of the ligament of Treitz, with a subsequent duodenojejunostomy with an Endo GIA stapler with 60 mm gold staples.

## Conclusions

WS is a rare pathology worldwide, which tends to have nonspecific symptoms such as a picture of high occlusion, so a high suspicion should be held, especially in patients who present certain risk factors, such as a history of previous surgeries or sudden weight loss, in order to offer treatment early and achieve an improvement in morbidity and mortality.

As there are no established criteria for surgical management, each surgeon must decide according to their judgment who benefits from an invasive procedure, individualizing each case and deciding per se which technique to use, the technique of choice being laparoscopic duodenojejunostomy, a procedure of broad complexity and which must be performed by surgeons with broad expertise.

## References

[1] Claro M, Sousa D, Abreu da Silva A, Grilo J, Martins JA (2021). Wilkie's syndrome: an unexpected finding. Cureus.

[2] Requena-López AA, Mata-Samperio BK, Cuadra-Reyes LA, Casillas-Vargas R (2020). Wilkie's syndrome as a cause of bowel obstruction in adults: a case report. Cir Cir.

[3] Güngörer V, Öztürk M, Arslan Ş (2023). A rare cause of recurrent abdominal pain; the coexistence of Wilkie's syndrome and nutcracker syndrome. Arch Argent Pediatr.

[4] Aranda Escaño E, Perfecto Valero A, Tellaeche de la Iglesia M, Fernández Gómez-Cruzado L, Santidrian Martinez JI (2020). Superior mesenteric artery syndrome (Wilkie syndrome): analysis of a series of 7 cases. Cir Esp (Engl Ed).

[5] Díaz-Martínez J, Hizojo-Aloé FT, Rivera-Chávez RJ, González-Hernández NA (2024). Misdiagnosed superior mesenteric artery syndrome due to diabetic gastroparesis. Case report and literature review. Int J Surg Case Rep.

[6] Rodriguez-Benites AF, Sanchez-Landers M, Deza Tarrillo NE (2024). [Wilkie's syndrome as a diagnostic challenge in intestinal obstruction: case report] (Article in Spanish). Rev Gastroenterol Peru.

[7] Lima Silva A, Antunes D, Cordeiro E Cunha J, Nogueira R, Fernandes D, Salazar T, Madureira Pinto C (2020). Epigastric pain and weight loss - a case of Wilkie's syndrome. Eur J Case Rep Intern Med.

[8] Pastén González A, Muñoz Araneda A, Peirano Bastías A, Rojas Castro S, Henríquez Alessandrini V (2016). [Superior mesenteric artery syndrome. A case report and review of the literature] (Article in Spanish). Cir Pediatr.

[9] Martínez H, Martínez S, Sánchez-Ussa S, Pedraza M, Cabrera LF (2019). Laparoscopic management for Wilkie´s syndrome. Cir Cir.

[10] Macías-Segura SA, Ávila-Bonilla AM, Rengifo-Alvis GA, Quintero-Pérez YY (2025). [Laparoscopic duodenojejunostomy for the management of Wilkie syndrome] (Article in Spanish). Rev Colomb Cir.

